# Enhancing the learning experience by empowering medical students to co-create learning tools and classroom activities

**DOI:** 10.5116/ijme.6702.4d43

**Published:** 2024-10-17

**Authors:** Parama Chaipackdee, Thanakrit Tanjararak, Parit Prechachaisurat, Bhranai Sammatat, Patomthan Marknui, Chalinee Monsereenusorn, Chanchai Traivaree, Wittawat Chantkran, Pasra Arnutti, Thammanoon Srisaarn, Ram Rangsin, Mathirut Mungthin, Dusit Staworn, Piya Rujkijyanont

**Affiliations:** 1 Phramongkutklao College of Medicine, Bangkok, Thailand; 2 Division of Hematology-Oncology, Department of Pediatrics, Phramongkutklao College of Medicine, Bangkok, Thailand; 3 Department of Pathology, Phramongkutklao College of Medicine, Bangkok, Thailand; 4 Department of Biochemistry, Phramongkutklao College of Medicine, Bangkok, Thailand; 5 Medical Education Unit, Phramongkutklao College of Medicine, Bangkok, Thailand

**Keywords:** Empowerment, pre-clinical students, student-centered learning, learning experience

## Abstract

**Objectives:**

This study aimed to enhance the learning experience among medical students by empowering them to co-create learning tools and classroom activities.

**Methods:**

A cross-sectional study was conducted with 10 participants from Year 2 of the new curriculum volunteering to participate in this study. Five were selected based on their diversities and empowered to design learning tools and class activities. Student satisfaction was presented as mean scores. A comparison of self-confidence scores in subjects learned before and after the class was analyzed using the paired t-test. Comparisons of multiple-choice question (MCQ) scores before and after the class between Years 2 (n = 96) and Year 3 of the previous curriculum attending inclass teaching (n = 98) were analyzed using the independent sample t-test.

**Results:**

A high level of satisfaction (M=87.5, SD=15.7%) and significant improvements in student self-confidence in subjects learned between before (M=46.4, SD=20.8%) and after (M=82.7, SD=16.9%) the class were noted (t _(223)_ = -23.73, p<.001). Additionally, Year 2 students achieved significantly higher MCQ scores after the class (M=85.6, SD=19.0%) compared with the scores from Year 3 (M=77.3, SD=23.6%) (t _(190)_ = 3.32, p<.001).

**Conclusions:**

Empowering medical students to co-create learning tools and class activities could positively enhance their learning experience. The result of this study addressed the importance of student empowerment with well-designed student-centered learning strategies based on their learning environment. Additional qualitative research is required to better understanding the “why” and “how” behind the findings of this study.

## Introduction

Student empowerment in classrooms is a strategy where students gain the ability and authority to make decisions and implement changes in their own learning path. Instead of doing what they are told or actively participating in their learning process, student empowerment gives the opportunity for students to invest more by creating their own learning journey. This powerful strategy encourages students to harmonize their academic knowledge, problem-solving skills, critical thinking, communication and collaboration which could ultimately reinforce them to develop grit and growth mindsets.^1^^-^^5^ In addition, the speed of social and cultural changes over the past decades has caused a paradigm shift in teaching and learning methods from traditional inclass didactic teaching where participating in class refers to staying quiet to active learning,^6^^-^^10^ in which the teaching and learning process mainly focuses on student interaction and engagement with learning activities using a variety of learning methods such as problem-based learning, questioning-based learning, game-based learning and student’s reflection. Moreover, the education field has evolved to the student-centered learning (SCL) method which focuses on providing learners the opportunity to choose and direct their own learning process based on their own knowledge, skills and self-interests^11^^-^^13^ as opposed to the long-established teacher-centered learning method which focuses primarily on the instructor who chooses learning topics and designs class activities.

Phramongkutklao College of Medicine (PCM) is the only medical cadet school in Thailand. It admits around 100 military cadets yearly and its six-year core curriculum is divided in three parts, namely, premedical year (Year 1) at the Faculty of Science, Kasetsart University, preclinical years (Years 2 to 3) at PCM and clinical years (Years 4 to 6) at Phramongkutklao Hospital. Given that PCM is a military college operated under the Ministry of Defense, it also conducts military training in addition to undergraduate education. In 2021, a major revision was made to our core curriculum of the Doctor of Medicine (MD) Program at our medical school to facilitate the vertical and horizontal integration of course contents in various disciplines. One of the changes involved the Hematopoietic and Lymphoreticular Systems Course (formerly named “Clinical Pathology Course”), which was moved from preclinical medical Year 3 to Year 2 in the new curriculum; therefore, the revision of the course learning strategy was essentially required to suit learning environment of Year 2 students who just started entering preclinical year, were new to the PCM environment where they had to stay at the dormitory during weekdays and were required to attend daily army basic training. According to our observation, physical and psychological stress was commonly found among Year 2 students which made many reluctant to answer questions and fully participate in class activity as well as falling asleep in class from tiredness. Our question was how to create an optimal teaching and learning strategy which could provide effective learning experiences by reducing stress and anxiety, encouraging students to focus more effectively on learning and fostering collaboration and teamwork among Year 2 students of the new curriculum.

To create an optimal learning strategy providing effective learning experiences, a pilot PCM student empowerment study was initiated in four classes of the Hematopoietic and Lymphoreticular Systems Course and to replace the teacher-centered learning method used in the same classes of the Clinical Pathology course of the previous curriculum. SCL was chosen as our main teaching and learning strategy and incorporated with the student empowerment approach during the learning process to provide students the authority to co-create their own course activities through identification of their learning goals and learning objectives followed by designing the course and selecting learning tools and class activities which were framed from their perspective. Although this approach has been previously implemented and shown to be effective in medical education^1^^,^^5^^,^^14^ it is new in Thailand and other low- and middle-income countries (LMICs) especially in preclinical years where inclass didactic teaching is still the main teaching method used. Herein, this student empowerment with well-designed SCL approach based on Year 2 students’ learning environment was conducted to affirm that this approach could be implemented in preclinical educational setting and potentially adapted in other institutions in LMICs. The objective of this study was to evaluate learning experience of the Year 2 students participating in this study by measuring their satisfaction, self-confidence towards subjects learned and knowledge gained during the class activities.

## Methods

### Study design and participants

A cross-sectional study was conducted among Year 2 preclinical medical students participating four classes of the Hematopoietic and Lymphoreticular Systems Course of the revised 2021 curriculum during the academic year 2022. The Royal Thai Army Medical Department Institutional Review Board granted permission to conduct the study.

Ninety-six Year 2 students of the revised 2021 curriculum attended the Hematopoietic and Lymphoreticular Systems Course in 2022. The announcement was made at one month before starting the class by asking for volunteers to codesign the course. Ten students volunteered to take part as representative students in this study, and five were selected based on their diversities in geographic residence, academic backgrounds, and grade point average scores to ensure that they represented the broader student body of the 2021 class. The role of class responsible teachers was to follow and support those five representative students throughout this study.

### Data collection and methods

The ADDIE model of instructional design^15^^,^^16^ was utilized to guide the design, development, implementation, and evaluation of our pilot PCM student empowerment study.

### Analysis and design of learning activity

Four classes of the Hematopoietic and Lymphoreticular Systems Course of the revised 2021 curriculum (hematopoiesis, approach to anemia, acute leukemia and hematopoietic stem cell transplantation) were selected for this pilot PCM student empowerment study. Five volunteering, representative students were invited to codesign the course activity.

A series of online meetings between representative students and class responsible teachers were conducted three weeks before the class. Representative students were informed regarding the course objectives and the specific aim of this study. They were encouraged to identify their learning goals and analyze their foreseen challenges during upcoming class. Potential learning methods were thoroughly discussed to suit their learning goals and overcome their challenges including the difficulty of course contents, the new and unfamiliar learning environment as well as exhaustion from daily army basic training which could result in falling asleep during class.

The representative students proposed using technology and digital games in learning activities given that they and their classmates were late adolescents and still enjoyed playing games. In addition, they thought that allowing students to review course materials before class would provide them free class time for fun activities which could prevent them and their classmates from falling asleep. After several meetings, the final decision was made from representative students to “gamify the flipped classroom using game-based learning”.

### Development of learning tools

Several types of game were discussed among representative students as the most sufficient learning tools to be used in their class activities. Finally, the representatives chose the Jeopardy game which could be downloaded as a free Jeopardy PowerPoint template from the “Slides Carnival” website. To develop the game, representative students were provided course materials including e-learning and clinical pathology textbooks two weeks before each class, in which the material contents were related to four assigned classes. After reviewing provided course materials, face-to-face and online meetings between representative students and class responsible teachers were conducted one week before each class. All five students brainstormed in creating questions to be used in the Jeopardy game. Different types of questions including multiple choice (MCQ), true or false, open-ended and cloze style, were created by students with the aim to make the game more fun during class activity. The level of the score (100 to 1000 points) was adjusted to the difficulty of questions. Throughout the learning tool development process, teachers only observed and verified the correctness and appropriateness of the questions.

### Implementation of learning activities

The remaining Year 2 preclinical medical students were informed to review course materials (e-learning and textbooks) according to their preferences at one week before each class. On the class day, the five representative students randomly divided their classmates in five groups and instructed them regarding the rules of the game. During the class activity, the Jeopardy game was solely conducted by the representative students and teachers only remained aside and facilitated learning for students. Points were accumulated from each class throughout the tournament. The team winner with the highest score was announced at the end of the last class, and ice cream treats were distributed to all students in appreciation.

### Research instruments

Two electronic assessment forms were the main research instruments used to collect data in this study. The forms were used to evaluate all Year 2 students’ satisfaction and self-confidence in subjects learned.  Student satisfaction was assessed at the end of each class using a simple numeric rating scale of 0 to 10, representing the lowest to highest satisfaction. Self-confidence towards subjects learned was assessed before and after each class using a simple numeric rating scale of 0 to 10, representing the lowest to highest self-confidence levels. In addition, an MCQ test was used to assess the capability of Year 2 students to gather data during class activity. Based on the Miller model for assessing competence,^17^ the MCQ test was designed and administered before and after each class and compared with the scores from the same questions obtained from Year 3 students of the previous curriculum attending in-class lectures. Each question had four choices and tested fact gathering or interpretation of the clinical or basic laboratory findings, e.g., “What is the rationale for autologous hematopoietic stem cell transplantation?” and “What is the diagnosis in a child with a history of exclusive breastfeeding and hypochromic microcytic anaemia?” To preserve privacy of all participating students and confidentiality of their data, the forms were anonymous and identifiable information of each individual was replaced with codes.

### Data analysis

Student satisfaction scores were analyzed and presented as mean with standard deviation (SD) or median (min-max). A comparison of self-confidence scores in subjects learned between scores obtained before and after the class was analyzed using the paired t-test. Comparisons of MCQ scores obtained before and after the class between Years 2 and 3, preclinical medical students were analyzed using the independent sample t-test. Using SPSS, and a p<.05 was considered statistically significant.

## Results

### Student characteristics

Ninety-six Year 2, preclinical medical students of the revised 2021 curriculum including the five representative students participated in the study. The age of students ranged between 18 and 21 years old. Males were more predominant than females at a ratio of 1.5:1 which was according to the desired number from the institution.

### Student satisfaction and self-confidence towards subjects learned

A median (min-max) of satisfaction score of all four classes among Year 2, preclinical medical students was 90 (10 to 100). Comparison of self-confidence scores in subjects learned among Year 2 students between scores obtained before and after the class was also performed revealing significantly higher self-confidence score after the class compared with the score before the class with mean scores of 82.7% (SD = 16.9) versus 46.4% (SD = 20.8), respectively (t_(223)_ = -23.73, p<.001).

In addition, several perspectives from three of five representative students were observed on their comments after the class activities. For example, subjects voiced, “I appreciate the teacher’s intention and effort to conduct this study to overcome all students’ challenges and to ensure that all students will have effective learning experiences”, “From all this, I think that I will be able to adopt my experience in composing questions from this project in preparing myself as well as my classmates for future exams in other classes”, “What I think I have done well is to design questions that cover most of the learning contents and the obstacle of this project is time constraints” and “What I think can be further developed is my assertiveness especially when speaking in front of the class and how to allocate time to study”.

### MCQ Assessment Comparison: Year 2 vs. Year 3 Med Students

**Figure 1:**
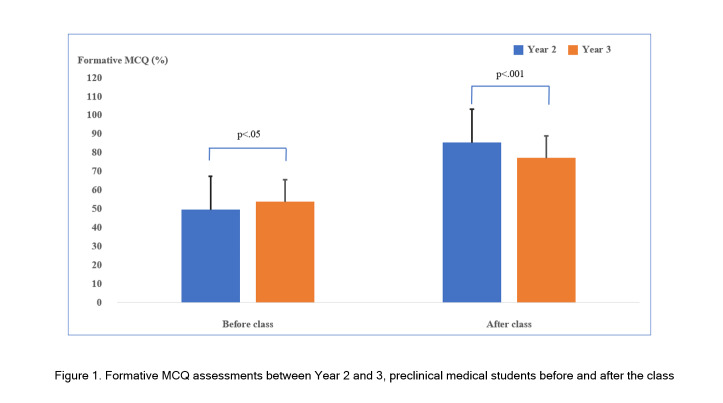
Formative MCQ assessments between Year 2 and 3, preclinical medical students before and after the class

To evaluate the capability of students to gather data during each class activity, MCQ was used to assess students before and after the class. Both Year 2 students in the new revised 2021 curriculum participating in this study and Year 3 students of the previous curriculum attending inclass lectures were tested using identical MCQs. MCQ scores obtained before the class between Year 2 and 3 students were compared revealing significantly higher scores among Year 3 students compared with the scores among Year 2 students with mean scores of 53.9% (SD = 21.0) versus 49.7% (SD = 24.8), respectively (t_(480)_ = -2.03, p<.05). Interestingly, after attending the class, Year 2 students achieved significantly higher formative MCQ scores compared with those of Year 3 students with mean scores of 85.6% (SD = 19.0) versus 77.3% (SD = 23.6), respectively (t_(190)_ = 3.32, p<.001) (Figure 1).

## Discussion

The current concept of medical education has shifted from a teacher-centered approach where teachers control the class to SCL^18^^,^^19^ in which students engage more concerning what and how they learn or even leading their own learning. SCL method have been increasingly used in medical education. To create SCL classes, input should be firstly obtained from students to identify their educational goals, and students should be encouraged to become leaders and decision-makers of their own learning including what materials they learn and how they learn it.^12^^,^^20^ In our study, SCL was selected as the main teaching/learning strategy in the Hematopoietic and Lymphoreticular Systems Course of the new curriculum and to replace the teacher-centered learning method used in the Clinical Pathology Course of the previous curriculum. To create our student-centered course design, a student empowerment approach was incorporated to SCL. Preclinical medical Year 2 students were empowered to choose their own learning and shape their own education based on their own interests and foreseen obstacles.^4^ This strategy benefits students in many ways including enhancing intrinsic motivation towards learning, self-esteem and soft skills in education. When students feel that their teachers allow them the freedom and responsibility to create their learning space, they are more likely to participate in class. This strategy can also help them develop self-determination, self-discipline and autonomy.^19^^,^^21^^, ^^22^

The learning environment is another key element supporting students' abilities to learn throughout their learning journey.^23^^-^^28^ A good learning method must be adaptable to different learning environments as well as groups of learners.^29^ For instance, we have used different methods for different levels of medical students in our institution such as using case-based learning among Year 4 medical students who are newly transitioning from preclinical to clinical learning environments but lack experience in caring for patients with different types of illnesses and self-directed learning among Year 6 medical students who need to spend more time with patients, make decisions on patient’s care and solve medical and nonmedical issues inside the ward. We believe that this learning method can promote students’ lifelong learning skills before they graduate to become doctors. To organize the Hematopoietic and Lymphoreticular Systems Course according to the revised 2021 core curriculum in our institution, we would like to establish a positive learning environment for our Year 2 medical students by empowering them to self-create and take ownership of their learning throughout this course. Empowering classrooms with a student-led learning approach was selected and incorporated to SCL with the goal to improve students’ learning experiences. This type of approach could also promote students’ collaborative learning environment as well as enhance their critical thinking and communication skills. The role of teachers was to only serve as facilitators and to provide access to appropriate and trustable resources (e-learning and textbooks).

Most Year 2 medical students were satisfied with this learning approach. This could be explained from some of their comments after each class regarding the gamifying flipped classroom learning style which allowed them the opportunity to review the lesson in advance and in the way they preferred (e-learning and/or textbooks), and the use of games to the class of late adolescents who are part of the digital generation which could also prevent them from falling asleep during the class activity. These findings resembled those of the study by Elzeky MEH and colleagues, in which gamified flipped classrooms improved nursing students' motivation, intensity of preparation, skills, knowledge, and self-confidence during laboratory clinical practice compared with the traditional flipped classrooms.^30^ In addition, the students felt more confident about the subjects they learned after the class. This could be from our SCL approach which encouraged them to identify their learning goals and take roles as leaders and decision-makers of their own learning activities.

To become the group winner, the group had to answer game questions correctly, so that each student had to exchange ideas with their friends in the group during class activity to overcome challenging questions. Students might have been self-motivated to extensively review the provided course materials before the class activity if they wanted to win the game. The more they prepared themselves before the class, the more confidence they would gain after finishing the class activity. These findings resembled those of the study by Canfield PJ and colleagues, in which SCL approach succeeded in providing students with confidence in analyzing laboratory data.^31^

Interestingly, the formative MCQ assessment scores among Year 2 students obtained after the class were significantly higher than the scores among Year 3 students attending traditional inclass didactic teaching, which reflected significant improvement of knowledge gained among Year 2 students during the class activity. This finding affirms the strength of our SCL approach with student empowerment being used during the learning process. According to these findings, teacher-centered learning should be substituted with SCL or student-led learning more in our current medical education practice.

According to the 2021 revised curriculum, all students were asked to write reflections in their portfolios of their intra- or extracurricular activities of interest and encouraged to provide anonymous comments. Several perspectives from three of five representative students were observed on their reflective writing in portfolios including appreciation, empathy, time management, self-awareness and self-motivation. These perspectives from representative students reflected their effective learning experiences and positive attitudes towards learning. The constructive feedback from class responsible teachers focused on showing them the benefits of “Learning how to learn”, encouraging them to apply that experience to other subjects and promoting their growth mindsets by emphasizing that their abilities and skills could be gradually developed through their diligence, effort and perseverance. These findings resembled those of the study by Centeio EE and colleagues, in which their “Believe in You Student Empowerment Program” revealed the potential to have a positive influence on students social emotional learning behaviors including self-awareness, self-management and relationship skills.^32^ Similarly, Vepraskas SH and colleagues reported that teaching inpatient bedside “presenter empowerment actions” during an interactive workshop among interns could increase their confidence.^33^ In addition, most comments from other participating Year 2 students expressed that they were satisfied, enjoyed the class activity and thought that this type of learning could encourage them and their classmates to remember the course contents better than traditional inclass lectures. Some students thought that questions from each class should be prepared by each group on a rolling basis instead of being prepared by only five representative students, and the group that prepares questions should lead the game during the class.

The limitations of this study included a small sample size of representative students and selected classes in the revised curriculum, which might have affected the results and not represent the whole student body class. The findings from the unique and specific populations (medical cadets) in this study might not be generally applicable among all medical students in Thailand. Moreover, we only used simple assessment methods (numeric rating scale and MCQ) to evaluate learning experiences of the students participating in our pilot study. Additional course evaluation models/frameworks are needed to further evaluate this course.^34^

## Conclusions

The SCL approach with student empowerment used during the learning process by allowing preclinical medical students to self-create and take ownership of their learning journey throughout the class activity resulted in high levels of student satisfaction. In addition, self-confidence towards subjects learned and significant improvement of knowledge gained during the class activity was observed compared with traditional inclass didactic teaching method. The result of this study provided essential clues that student empowerment with well-designed SCL strategies based on students’ learning environment is vital for preclinical medical education, in which this approach could be implemented in LMICs where traditional inclass didactic teaching remains the main teaching method used. Allowing preclinical medical students to create their own learning space could enhance their learning experience, strengthen their self-confidence towards subjects learned and improve their attitude towards learning. Further qualitative research is needed to gain a deeper understanding of the reasons and mechanisms behind the findings of this study.

### Acknowledgements

The funding from the Phramongkutklao College of Medicine and Phramongkutklao Hospital, Royal Thai Army was used to conduct the study, analyze, interpret the results and submit for publication.

### Conflicts of Interest

The authors declare that they have no conflict of interest.
